# Editorial: Insights in thrombosis: 2022

**DOI:** 10.3389/fcvm.2023.1240626

**Published:** 2023-07-06

**Authors:** Jonathan Douxfils, Hugo ten Cate, Roberto Pola

**Affiliations:** ^1^ Unité de Recherche en Pharmacologie Clinique et Toxicologie, Département de Pharmacie, Faculté de Médecine, Université de Namur, Namur, Belgium; ^2^ QUALIblood s.a., QUALIresearch, Namur, Belgium; ^3^ Thrombosis Expertise Center and Carim School for Cardiovascular Diseases, Maastricht University Medical Center, Maastricht, Netherlands; ^4^ Section of Internal Medicine and Thromboembolic Diseases, Department of Medicine and Geriatrics, Fondazione Policlinico Universitario A. Gemelli IRCCS, Università Cattolica del Sacro Cuore, Rome, Italy

**Keywords:** direct oral anticoagulant (DOAC), venous thrombembolism, arterial thromboembolic event (ATE), factor v leiden, factor XI inhibitors, antithrombotic, antiplatelet therapy

**Editorial on the Research Topic** Insights in thrombosis: 2022

Venous and arterial thromboembolism are common, multi-causal diseases with serious short and long-term complications. Although therapies exist to treat or diminish the occurrence and recurrence of these thrombotic events, current strategies are not optimal as thromboembolic disease remains one of the top leading causes of death worldwide (1). Venous thromboembolism (VTE) has a high mortality rate in the first year, especially within the first 30 days, and has a risk of recurrence which may depend on genetic risk factors. This Research Topic “Insight in Thrombosis: 2022” aimed at shedding light on the progress made in the past decade in the thrombosis field. As of today, it has already generated more than 10,000 views, revealing the interest in this important medical field.

In this special edition, Eppenberger et al. provided a systematic literature review and meta-analysis to investigate whether heterozygous factor V Leiden patients are at increased risk of VTE recurrence compared to those without this mutation. Among the 24 studies included in their analyses, they find that heterozygous FVL increases the risk of recurrence by 46% (95% CI, 31%–64%). As the 5-year incidence of VTE recurrence is around 20%–25%, a 46% increased risk could raise the incidence of recurrent events by 9%–12%. Although there is a current debate in the literature regarding the need for FVL testing, or for any other thrombophilic trait, after a first VTE event, more efficient algorithms including such risk factors are certainly needed to better predict the risk of VTE recurrence and to guide patient management.

Besides genetic predisposition, cancer is also a leading cause of thromboembolic events (2). Cancer patients also share a higher rate of VTE recurrence and are also exposed to a higher risk of bleeding while on antithrombotic therapy. In these otherwise carefully monitored patients, incidental VTE is common and is sometimes regarded as similar to symptomatic VTE. However, few data are available in the literature to support these assumptions and patients may benefit from more appropriate, individualized, management since recurrence, bleeding, and death may not be similar between the two groups. Barca-Hernando et al. perform a *post hoc* analysis of prospective studies of cancer patients with VTE that occurred between 2008 and 2019. They find that cancer patients with incidental VTE and symptomatic VTE had similar rates of bleeding and VTE recurrence in long-term follow-up (i.e., up to 10 years), supporting the current recommendations that incidental VTE patients should be treated similarly to the symptomatic group. However, at 6-month follow-up, the rate of bleeding events was significantly higher and VTE recurrence rates were lower in the incidental VTE cancer patients meaning that a more tailored approach could be proposed in these patients, at least during the first 6 months after the VTE event. Nevertheless, multiple confounders may bias the analysis and future studies are thus needed to assess which patients within the incidental VTE group may require a reduced dosage and/or limited duration of anticoagulation therapy.

In the quest for the Holy Grail of antithrombotic therapy, factor XI inhibitors may be an even more promising class of drugs than direct oral anticoagulants (DOACs). Nopp et al. review the current rationale and evidence for the use of this new class of agents in the prevention and treatment of VTE. In their article, they highlight unmet clinical needs of anticoagulation therapy, lay out the rationale and evidence for inhibiting FXI, discuss FXI inhibitors in current clinical trials, and provide an outlook on the potential clinical application of these novel anticoagulants. They further conduct an exploratory meta-analysis of phase II studies assessing the efficacy and safety of factor XI inhibitors compared to enoxaparin for the prevention of VTE in patients undergoing total knee arthroplasty. Altogether, IONIS-FXI-Rx, osocimab, abelacimab, and milvexian reduced the risk of VTE by 41% and the risk of bleeding by 59% compared to enoxaparin. Besides their use in major orthopedic surgery, these new therapeutics may also be valuable in patients with end-stage kidney disease and in patients with atrial fibrillation in light of the reassuring safety and pharmacological data that supported their progress to phase III studies. Nevertheless, some studies also reported that higher intensity of FXI inhibition could impair hemostatic function questioning their position as drugs with a more favorable bleeding risk profile. In addition, in most of the targeted applications, FXI inhibitors will need to be compared to DOACs which have already been widely implemented in clinical practice. Results from ongoing phase-III clinical trials are awaited to guide the potential transition from current anticoagulants to FXI inhibitors for specific indications.

Combining anticoagulant and antiplatelet agents may also improve patient outcomes as highlighted by the combination of rivaroxaban with acetylsalicylic acid (ASA) for the treatment of peripheral arterial disease (PAD). In their pilot study, Jurk et al. evaluate the platelet activation status *in vivo* and platelet reactivity *in vitro* in platelet-rich plasma (PRP) from patients with PAD receiving ASA before endovascular revascularization (EVR), ASA plus clopidogrel after EVR, and ASA plus rivaroxaban during a long-term follow-up. They find that the addition of rivaroxaban reduces the thrombin propagation phase of platelet CD36-sensitive thrombin formation in patients with PAD treated with ASA plus rivaroxaban compared to ASA monotherapy, which is more pronounced than during ASA plus clopidogrel therapy. Their data also reveal that rivaroxaban moderately inhibits platelet activation mediated by the thrombin receptor PAR-1, but not by thrombin, confirming the intrinsic link between platelets and coagulation. Although already clinically demonstrated, combined inhibition of selected targets could be of added value in many patients with cardiovascular disease where the etiology involves both coagulation and platelet aggregation, provided the bleeding risks are acceptable.

Although new therapeutic strategies and diagnostic algorithms are cornerstones for patient management, adherence to current guidelines is also mandatory to ensure the best outcome at the patient level. Mertins et al. evaluate adherence to current guidelines for secondary prevention of VTE from a large prospective cohort of 6,243 patients. The adherence to the current guidelines was only 36.1%, with overtreatment representing the main type of non-adherence. Interestingly, adherence to the guidelines was not associated with lower mortality, hospitalization, admission to nursing homes, or cost even after adjustment for confounders. On the other hand, the Prognostication in acute Pulmonary Embolism (IPEP) trial showed that prognostication and use of objective criteria for mobilization and early hospital discharge are safe and associated with a reduction in downstream laboratory or echocardiographic testing. It is also effective in reducing the length of hospital stay (LOS) by 2 days, compared with usual care. In a *post-hoc* analysis of this IPEP trial, Jiménez et al. show that the use of the simplified Pulmonary Embolism Severity Index (sPESI) is effective in reducing LOS both in the low- and intermediate-high-risk subgroups. Interestingly, this score also identified a significantly higher proportion of patients at intermediate-high risk for short-term complications. This is particularly important since approximately 5% of these patients might deteriorate after diagnosis and initiation of therapy and require monitoring over the first hours or days.

In the general population, there is also a need to prevent or delay the occurrence of thromboembolic events and a part of this solution can be found in our alimentation. Rautenbach et al. elucidate the effect of 25-OH vitamin D on the γ’ splice variant of fibrinogen and fibrin clot characteristics. They find that 25-OH vitamin D modulated fibrinogen γ’ and the maximal clot absorbance. According to the authors, the clinical significance of their findings is questionable and remains to be determined. However, as vitamin D supplementation may downregulate the expression of cytokines and is beneficial in glycemic control, it may influence clot properties by lowering the level of fibrinogen or changing the glycation of the activated fibrinogen molecules, making it less resistant to the degradation y plasmin. Knowing the potential role of vitamin D in reducing cardiovascular risk, future intervention trials in which responsible exposure to sunlight, the inclusion of vitamin D-rich foods, and/or whether supplementation or fortification with vitamin D analogs are explored in relation to blood coagulation, should be designed to investigate novel therapeutic options for those with a high risk of developing cardiovascular disease.

Finally, there is a need to consistently improve our knowledge of the complex enzymatic cascade which characterizes hemostasis and inflammatory and complement pathways. Humphreys et al. highlight the current understanding of the physiological role of serine protease inhibitors (SERPINs) in hemostasis and inflammatory disease progression, with emphasis on the fibrinolytic pathway, and how this becomes dysregulated during disease. The SERPINs *α*2-antiplasmin, plasminogen-activator inhibitor-1 (PAI-1), plasminogen-activator inhibitor-2, protease nexin-1, and C1-inhibitor play crucial inhibitory roles in the regulation of the fibrinolytic system and inflammation. Elevated levels of these SERPINs are associated with an increased risk of thrombotic complications, obesity, type 2 diabetes, and hypertension. Conversely, deficiencies of these SERPINs have been linked to hyperfibrinolysis with bleeding and angioedema. These SERPINs may serve for diagnosis purposes but are also the target for therapeutic action as *α*2-antiplasmin, PAI-1, and C1-inhibitor have been associated with a variety of thromboembolic and inflammatory conditions. Further research in this field is required to provide innovative strategies that may deal with complex settings such as immunothrombosis, thrombolytic therapy, and even in obstructive sleep apnea (3).

This Research Topic reveals that the field is still evolving by providing a more tailored and individualized management of the patients although it has already radically changed since the advent of DOACs, which have improved the efficacy, safety, and adherence to anticoagulant therapy. Whether from genetics, patient management, prevention, drug combinations, or new target discovery, the battle against thromboses is not yet won and these areas deserve specific attention from the scientific community. This is even more important in the context of an aging population and risk factors are more prevalent due to our sedentary style of life.
